# Oxygen-Related Differences in Cellular and Vesicular Phenotypes Observed for Ovarian Cell Cancer Lines

**DOI:** 10.5772/62219

**Published:** 2016-01-22

**Authors:** Evo K. Lindersson Søndergaard, Lotte Hatting Pugholm, Rikke Bæk, Malene Møller Jørgensen, Anne Louise Schacht Revenfeld, Kim Varming

**Affiliations:** 1 Department of Clinical Immunology, Aalborg University Hospital, Aalborg, Denmark

**Keywords:** Extracellular Vesicles, Ovarian Cancer Cell Line, EV Array, Phenotype, Hypoxia

## Abstract

Extracellular vesicles (EVs) are one of several tools that cells use to communicate with each other. This communication is facilitated by a number of surface-associated proteins and the cargo of the vesicles. For several cancer types, the amount of EVs is observed to be up-regulated in patients compared to healthy individuals, possibly signifying the presence of an aberrant process. The hypoxia-induced release of EVs from cancer cells has been hypothesized to cause the malignant transformation of healthy recipient cells.

In this study, the phenotype of cells and EVs from the ovarian cancer cell lines, COV504, SKOV3, and Pt4, were quantified and analysed under normoxic and hypoxic conditions. It was shown that both cells and EVs express common markers and that the EV phenotype varies more than the cellular phenotype. Additionally, cells subjected to 24 hours of hypoxia compared to normoxia produced more EVs.

The phenotyping of EVs from cancer cell lines provides information about their molecular composition. This information may be translated to knowledge regarding the functionality of EVs and lead to a better understanding of their role in cancer.

## 1. Introduction

The interest in extracellular vesicles (EVs) has increased immensely over the last few years. EVs are believed to be an important component of extracellular communication [1], although, their exact functions are not fully understood. In humans, EVs are a heterogeneous population of membrane-enclosed vesicles that are released into the extracellular space by most cell types. They have been shown to be involved in a variety of important physiological and immunological processes [2–4]. They may also be involved in the progression of pathological conditions such as cancer [5,6]. EVs are often divided into three major subpopulations based on their size, biogenesis and molecular composition, namely exosomes, microvesicles and apoptotic bodies [1,7–11]. Even though the molecular composition of these three subsets of EVs is different, several markers overlap. These markers are not ubiquitously expressed on EVs but are found on a majority of them [7,9,12]. So far, identification of a specific marker that, with certainty, can distinguish between the three subsets has not been discovered. Along with these proteins, the molecular composition depends on the cellular source, often mirroring the parent cell [13,14]. Notably, recent data suggest that the phenotype of EVs is specific and well-regulated, and not just the result of a casual sampling of molecules from the parent cell [15]. The current study investigates the subset of EVs that presents CD9, CD63 and CD81.

A large number of studies have reported that EVs are involved in the development and progression of cancer. Their role in cancer is emphasized by the fact that the release of EVs is accelerated in tumour cells, as demonstrated by *in vitro* studies, as well as by the large amount of EVs or EV-like structures that can be purified from plasma, ascites and pleural effusions of cancer patients [16–18].

Tumour-derived EVs have been reported to both stimulate and suppress tumour-specific and non-specific immune responses. This capacity may be explained by the similarity of the protein composition of EVs and the parent cell type, which suggests that the tumour-derived EVs contain tumour-specific antigens that can stimulate or inhibit an anti-tumour response [17–22].

Hypoxia is one of many factors that are believed to be important for the maintenance of the tumour milieu. Hypoxic regions are observed in most solid tumours [23,24] and numerous factors involved in the promotion of metastasis have been described to be induced by hypoxia [25–28]. Furthermore, it has been reported that patients with hypoxic primary tumours developed more metastasis than patients with less hypoxic tumours [29,30]. Hence, hypoxia is an important factor for the tumour milieu, as well as for the metastatic processes. Taken together, it seems that both hypoxia and tumour-derived EVs can play important roles at multiple stages of tumour pathogenesis, ranging from suppressing the anti-tumour responses to facilitating the formation of a suitable microenvironment in distant metastatic sites [31–35].

In this study, the cellular and EV phenotypes from the three ovarian cancer cell lines, COV504, SKOV3 and Pt4, were analysed. These three cell lines were chosen as they all originally derive from ovarian cancer patients with different tumour forms. Additionally, it was investigated whether hypoxia could affect the phenotypes of cells and EVs. For the cellular phenotype, a flow cytometric analysis was used and five different markers were chosen on behalf of their functional differences. Carboxic anhydrase IX (CAIX) and Carboxic anhydrase XII (CAXII) are known as hypoxic markers [36]. CD151 is known as an exosomal protein associated with tumour progression. It enhances cell motility, invasion and metastasis of cancer cells and is over-expressed in many tumour types [37]. CD9 and CD81 are considered as general exosomal markers [9,12]. CD9 is a cell surface glycoprotein known to complex with, e.g., integrins and other tetraspanins. It can modulate cell adhesion and migration and trigger platelet activation and aggregation [38]. CD81 is a surface glycoprotein that is known to complex with integrins, and it is involved in activation, co-simulation and differentiation [39]. For the EV characterization, the protein microarray based EV Array technique was applied to analyse and phenotype a subset of these cell-derived vesicles, which carries the general EV markers, CD9, CD63 and CD81 [40]. The extensive phenotyping involved 31 markers that are related to general EV proteins, cell-specific markers and a number of cancer markers.

## 2. Materials and methods

### 2.1 Cell Cultures

SKOV3 (ATCC® HTB-77™; ATCC, Manassas, VA, USA) and Pt4 (primary cell line from ascites from an ovarian cancer patient) were cultured in RPMI-1640 (Gibco, Life Technologies, CA, USA) and supplied with 10% heat-inactivated foetal calf serum (FCS) (Gibco), 100U/ml penicillin and 0.1mg/mL streptomycin (Amplicon, Odense, DK). COV504 (07071902-1VL, Sigma-Aldrich, St. Louis, MO, VA) was cultured in Dulbecco's Modified Eagle's Medium (DMEM) (Gibco) supplemented with 10% FCS, 100U/ml penicillin and 0.1mg/mL streptomycin.

The cells were cultured in 96-well plates in triplicates, with a concentration of 1.25 × 10^5^ cells/well in a total volume of 250μl. Media controls were included for each cell line. Before supplementing the media with FCS, the FCS was centrifuged at 100.000 x g for 24h, at 4°C (Ti45 rotor, Beckman Coulter, Brea, USA) to deplete the EVs present in the FCS. Under normoxic conditions, the cells were cultured at 37°C in 5% (v/v) CO_2_ and with an atmospheric O_2_ concentration. To induce hypoxia, the cells were cultured at 37°C in 5% (v/v) CO_2_ and 1% (v/v) O_2_.

To determine the cell density, images of the different cell lines were captured for each experimental condition, using a FLOID Imaging Station (Life Technologies).

### 2.2 Phenotyping Cells by Flow Cytometry

The adherent cells were detached with trypsin (0.25%/EDTA) (Gibco) for 10 min. For each cell line, cells from the triplicate wells were pooled and washed once in cell media and then twice in PBS (500 x g, 5min, room temperature (RT)).

The following antibodies and reagents were used for flow cytometry: Anti-CD151 (210127) and anti-CAXII (315602) (R&D Systems, Minneapolis, MN, USA); 7-Aminoactinomycin D (7AAD), anti-CD9-PerCP Cy5.5 (M-L13), control mouse IgG1(k)-PerCP-Cy 5.5 (MOPC-21) and mouse IgG1(k)-PE (MOPC-21) (BD Bioscience, San Jose, CA, USA); Anti-CAIX (2D3) (Abcam, Cambridge, MA, USA); anti-CD81-PE (1.3.3.22) (Ancell Corporation, MN, USA); and, as a secondary antibody, goat anti-mouse IgG1(k)-PE (DakoCytomation, Glostrup, DK).

For antibody staining, each sample contained 1.2 × 10^5^ cells. The cells were mixed with the relevant antibodies and incubated for 30 min at RT in the dark. The cells were then washed twice with PBS-BSA (0.5%, w/v) (500 x g, 5min, RT). The cells that had incubated with unconjugated antibodies were mixed with goat anti-mouse-PE and subsequently incubated for 30 min at RT in the dark. Afterwards, the cells were washed, resuspended in sheath fluid with 1% paraformaldehyde (BD Bioscience) and analysed.

The acquisition of the stained cells was performed on a FACSCanto II using FACSDiv™ software (version 6.1.3, BD Biosciences). The analysis of the data was carried out with the FlowJo software (version 10.0.7, FlowJo LLC, Ashland, OR, USA). The cellular phenotype for each marker was determined for all events except cell debris by applying a FSC/SSC gate that excludes debris. The negative isotype controls were utilized to identify the positive events. For all populations, the median fluorescence intensity (MFI) was the statistical value of choice.

### 2.3 Preparation of EVs from Cell Cultures

For preparation of the EVs, the harvested cell culture supernatant was centrifuged at RT for 10 min at 700 x g to pellet the cells. The cell-free EV supernatants were supplemented with protease inhibitors (Complete, EDTA-free, Roche, DE, USA) and stored at −40°C until an analysis on the EV-Array was carried out. There was no further purification of the EVs prior to measuring on the EV Array.

### 2.4 Phenotyping Vesicles by the EV Array

Microarray printing was performed on a SpotBot® Extreme Protein Edition Microarray Printer with a 946MP4 pin (ArrayIt, CA, US). As positive and negative control, 100 μg/mL of biotinylated human IgG and PBS with 5 % glycerol was printed, respectively. Epoxy coated slides (75.6 mm × 25.0 mm; SCHOTT Nexterion, DE) were used and then left to dry at RT overnight prior to further analysis.

For the EV Array, the following antibodies and protein were used for capture: Annexin V (polyclonal), CAXII (315602), CD13 (498001), CD82 (423524), CD142 (323514), CD151 (210127), LAMP2 (H4A3), MUC1 (604804), TNF RI (16803), TNF RII (22210), Tspan 8 (458811) (R&D systems); AKAP3 (C-20), EpCam (O.N.277), Mucin 16 (X306), NY-ESO-1 (E978), PLAP (8B6), TLR3 (TLR3.7) (Santa Cruz Biotechnologies, TX, USA); Alix (3A9), CD63 (MEM-259), CD309 (7D4-6), HLA ABC (W6/32) (Biolegend, CA, USA); CAIX (2D3), Flotilin 1 (polyclonal), TSG101 (5B7), sTn (219) (Abcam); CD9, and CD81 (LifeSpan BioSciences, Inc. Seattle, WA, USA); EGFR (polyclonal), EGFRvIII (polyclonal) (Antibodies online.com (DE)); CD171 (polyclonal) (Sigma-Aldrich); ICAM-1 (R6.5) (eBiosciences, CA, USA). The bovine protein Lachtadhedrin (Haematologic Technologies Inc, Vermont, USA) was used for capture along with the listed antibodies. The antibodies and the protein were printed in triplicates at 90 – 200 μg/mL diluted in PBS containing 5% glycerol.

For the semi-quantification of EVs, the antibodies, CD9, CD81 (LifeSpan Biosciences, Inc.) and CD63 (MEM-259) (BioLegend), were printed in 18 repeated spots in a mixture/cocktail of 100 μg/mL of each antibody diluted in PBS and containing 5% glycerol.

For detection, a cocktail of the following biotinylated antibodies were used: anti-CD9, -CD63 and -CD81 (LifeSpan BioSciences, Inc.).

The catching and visualization of EVs, as well as the data analysis, were performed as previously described in [40] with minor modifications. In short, the slides were blocked (50 mM ethanolamine, 100 mM Tris, 0.1% SDS pH 9.0) prior to incubation with EV-containing cell supernatants (100μl for phenotyping and 75μl for the semi-quantification). The incubation was performed in Multi-Well Hybridization Cassettes (ArrayIt, CA, USA) at RT for two hours, followed by overnight incubation at 4 °C. After washing (PBS with 0.05% Tween®20), the slides were incubated with biotinylated detection antibodies (anti-CD9, -CD63, and -CD81) (1:1500 in wash buffer). After washing, 30 min incubation with Cy5-labelled streptavidin (Life Technologies) (1:1500 in wash buffer) was performed for detection. Prior to scanning, the slides were first washed in washing buffer, followed by MilliQ water, and dried using a Microarray High-Speed Centrifuge (ArrayIt). Scanning and spot detection were performed as previously described [40]. Briefly, the intensity of the antibody signal was calculated by subtracting the mean of the background (without sample/blank) from the mean of the triplicate antibody spots. This signal was then divided by the signal from the mean of the triplicate negative spots (without capture antibody, with sample). The relative fluorescence intensity was subsequently log2 transformed.

Graphs and statistics were made in GraphPad Prism (ver. 6.04, GraphPad Software, Inc. CA, USA) and Excel (ver. 2013, Microsoft, USA) and heatmaps were generated in Genesis (ver. 1.7.6, IGB TU Graz, Austria). An unpaired t-test was applied to test for differences between the semi-quantitative measurements of EVs from the same cell line subjected to two different conditions. Differences between groups were considered statistically significant when *p* < 0.05. Unless otherwise specified, the data are presented as mean ± SD.

## 3. Results

### 3.1 Cell Proliferation and Viability Following Hypoxic Incubation

The three cancer cell lines, SKOV3, Pt4 and COV504, were incubated under normoxic or hypoxic conditions. The cell number and viability were determined for each cell line directly after thawing, and after 12 and 24 hours of culturing (Table I). To investigate the oxygenic state of the cell cultures, the cell lines, SKOV3 and COV504, were used as examples and the expression of the glucose transporter, GLUT1, and hypoxia-inducible factor, 1α (Hif-1α), were analysed after culturing the cells under hypoxic or normoxic conditions for 24 hours. Data are presented in the supplementary (S1). The proliferation and the viability of SKOV3 and Pt4 did not change much over time or between normoxic and hypoxic conditions. On the contrary, COV504 showed a decrease in the viability under hypoxic conditions, whereas the normoxic population retained a higher viability after 24 hours. Furthermore, the cell count of COV504 following 24 hours of incubation under hypoxic conditions was only half the cell count observed in normoxic conditions.

**Table 1.** Listed are the percentages of cell death (positive for 7AAD), percentages of cell density and cell count following harvest at the different time points and conditions (normoxia and hypoxia) for the three ovarian cancer cell lines SKOV3, COV504 and Pt4

Cell Line	Tissue	Time Point	7AAD Normoxia	7AAD Hypoxia	Cell Density Normoxia	Cell Density Hypoxia	Cell Count Normoxia	Cell Count Hypoxia
SKOV3	Adenocarcinoma ovary, ascites, epithelial	0h	8%	8%	—–	—–	125000	125000
		12h	8%	13%	50%	50%	103750	115000
		24h	11%	8%	100%	100%	140000	130000
		48h	10%	12%	100%	100%	163750	141250
COV504	Epithelial serous carcinoma, pleural effusion, epithelial ovary	0h	1%	1%	—–	—–	125000	125000
		12h	20%	25%	30–40%	30–40%	62500	60000
		24h	11%	29%	70–85%	70–80%	107500	52500
Pt4	Serous papillary carcinoma ovary, stage 2, ascites	0h	4%	4%	—–	—–	125000	125000
		12h	13%	19%	50–60%	60–70%	117500	97500
		24h	14%	14%	60–80%	70–80%	132500	122500

### 3.2 Cellular Expression of Selected Surface Markers

**Figure 1.**
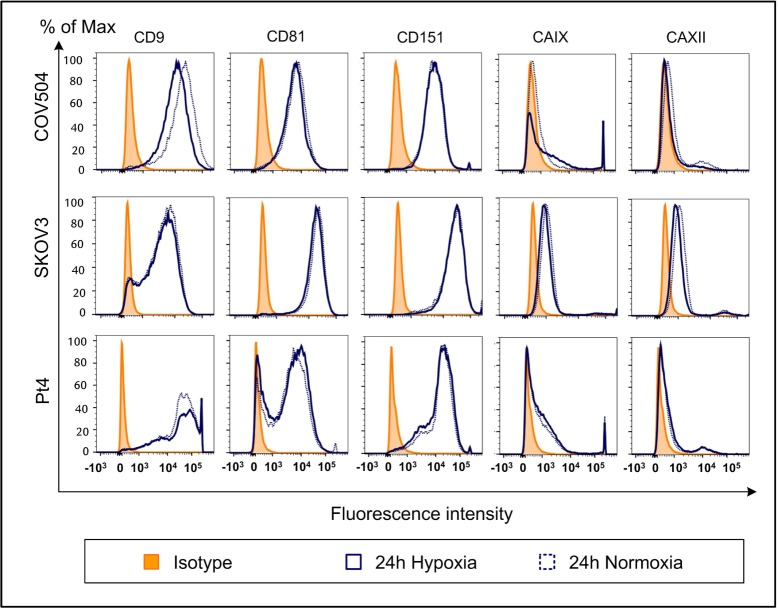
The influence of hypoxia on the cellular phenotype of ovarian cancer cell lines. The different cell subsets were stained with antibodies against CAIX, CAXII, CD151, CD81, CD9 and matched isotype controls and were analysed by flow cytometry. The overlays of the expression displayed by the cells subjected to either normoxic or hypoxic conditions for 24 hours for the three cell lines, COV504, SKOV3 and Pt4, are displayed.

The expression of CD9, CD81, CD151, CAIX and CAXII were evaluated for the three cell lines, COV504, SKOV3 and Pt4, followed by incubation at normoxic or hypoxic conditions. The specific surface expression was determined by flow cytometry directly following thawing after 12 and after 24 hours. The expression of the five markers changed marginally over time but, since most cultured cells need time to adapt to changes and to a new environment, the time point 24 hours is illustrated (Figure 1). The flow cytometric analysis showed that the three cell lines clearly expressed CD9, CD81 and CD151. SKOV3 also expressed CAIX and CAXII (> 3 times the isotype MFI), whereas COV504 and Pt4 expressed CAIX and CAXII (< 2 times the isotype MFI). In addition, the effects of hypoxia on the expression of the five cell surface markers were investigated (Figure 1). After 24 hours of hypoxic conditions, the median CD9 expression by COV504 was only half the MFI of cells cultured under normoxic conditions, indicating that CD9 expression by COV504 was affected by hypoxic conditions. For all other markers, the expression on COV504 remained unaffected by hypoxic conditions. Similarly, the expression of the five markers on SKOV3 and Pt4 were unaffected by hypoxic conditions.

### 3.3 Phenotyping of Cell-derived EVs

**Figure 2.**
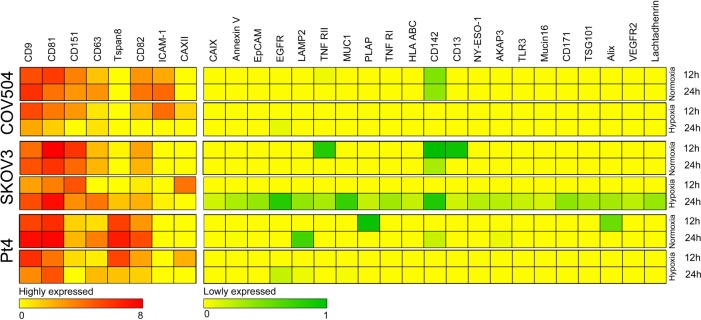
Phenotyping of EVs from ovarian cancer cell lines. The EV phenotypes were profiled using the EV Array printed with 31 different capture antibodies and detected with a cocktail of antibodies against CD9, CD63 and CD81. The heatmaps of the phenotyping of EVs derived from COV504, SKOV3 and Pt4 subjected to either normoxic or hypoxic conditions for 12 or 24 hours.

In addition to the cellular phenotypes, the phenotypes of the cell-derived EVs found in the cell free supernatants were determined. The EVs captured and measured by the EV Array had a size of 30–300 nm (supplementary Figure S2). This extensive phenotyping involved 31 markers comprising general EV proteins, cell-specific markers and a number of cancer markers. The effect of the oxygen levels of the EV phenotype was evaluated over time. A summary of the EV phenotypes can be seen in Figure 2. The heatmaps demonstrate the phenotype of EVs from the three ovarian cancer cell lines, subjected to either normoxic or hypoxic conditions for 12 or 24 hours. Phenotypically, the EVs from the three cell lines resembled each other and displayed more or less the same markers under normoxic conditions. The EVs derived from all three cell lines grown under normoxic conditions expressed CD9, CD81, CD151, CD63, Tspan8, CD82, ICAM-1, CAXII and CD142, whereas 16 markers were not detected. Only six markers (LAMP2, TNF RII, PLAP, CD13, AKAP3 and Alix) showed a variable expression. On the contrary, there were phenotypical differences between the EVs derived from cells subjected to hypoxic conditions. The EVs from COV504 and Pt4 had a tendency to express their markers most strongly after 24 hours in normoxic conditions, whereas the EVs from SKOV3 expressed their markers more strongly during hypoxic conditions. In particular, this was observed with CD9, CD81, CD151, CD63 and CD82. Furthermore, the EVs from SKOV3 expressed considerably more markers following 24 hours of hypoxia compared to the EVs from COV504 and Pt4. When comparing the expression level after only 12 hours, COV504 and Pt4 expressed their markers most strongly under hypoxic conditions, whereas SKOV3 expressed most of the markers most strongly under normoxic conditions. Notably, when looking at the three tetraspanins, CD9, CD63 and CD81, the EVs derived from all three cell lines did not express CD63 as strongly as the other two known EV markers.

### 3.4 Comparison of EV and Cellular Phenotypes

**Figure 3.**
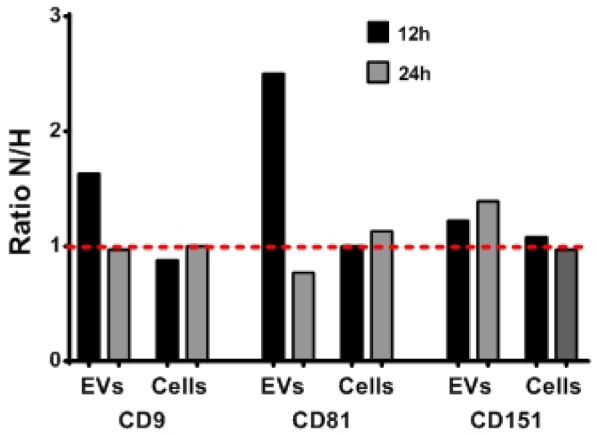
Variations in EV and cellular phenotypes. The cellular phenotype was analysed by flow cytometry, whereas the phenotype of the EVs was determined with EV Array. The graphs display the ratio of the phenotypical variations of the three markers, CD9, CD81 and CD151, between the EVs (mean values) and cells (median values) from the cell line, SKOV3, after either 12 or 24 hours under normoxic (N) or hypoxic (H) conditions. There is one mean value (EVs) or median value (cells) for each of the three markers. Therefore, no statistical measurements were performed.

In order to investigate whether the EV phenotype mirrored the cellular phenotype, a comparison of the protein composition was made. SKOV3 is used here as an example but the other cell lines showed similar results (data not shown). Figure 3 illustrates the relation between the cellular and EV phenotypes (for the three markers, CD9, CD81 and CD151) under normoxic and hypoxic conditions. The amount of CD9, CD81 and CD151 presented on the EVs varied if the cells were subjected to normoxic compared to hypoxic conditions. The EVs displayed more pronounced CD9 and CD81 after 12 hours of cell incubation in normoxic conditions compared to hypoxic conditions, whereas, after 24 hours, the presentation of CD9 was almost the same under both conditions and CD81 was slightly more expressed under hypoxic conditions. CD151 was more presented on the EVs under normoxic compared to hypoxic conditions for both 12 and 24 hours. On the contrary, there were small differences in the cellular expression of CD9, CD81 and CD151 observed over time with the two conditions, but the differences were not as defined as the ones observed for the corresponding EVs.

### 3.5 The Production of EVs is Influenced by Various Factors

**Figure 4.**
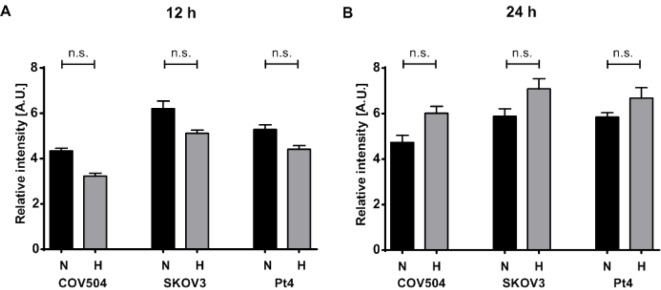
The EV production is influenced by several factors. The amount of EVs was semi-quantitatively estimated with the EV Array measuring the total fluorescence signal when the EVs were captured, as well as detected with a cocktail of antibodies against CD9, CD63, and CD81. Each bar represents the relative fluorescence intensity (mean ± SD) of each triplicate measurement on the EV Array. The relative amount of EVs produced by COV504, SKOV3 and Pt4 under either normoxic (N) or hypoxic (H) conditions for 12 hours (A) and for 24 hours (B) is displayed.

The EVs from the cell culture supernatants were harvested at different time points and the relative amount of EVs were estimated with the EV Array. This was achieved by measuring the total fluorescence signal when antibodies against the exosomal markers (CD9, CD63 and CD81) were used both for the capture and detection of the EVs. Figure 4 displays the semi-quantitative measurement of the relative amount of EVs derived from cells after 12 hours (A) and 24 hours (B) for all three cell lines subjected to either normoxic or hypoxic conditions. After 12 hours, the highest amount of EVs was detected from the cells grown under normoxic conditions, which was observed for all three cell lines. In contrast, after 24 hours, the relative amount of EVs considerably increased under hypoxic conditions.

From 12 hours to 24 hours, the relative amount of EVs released by COV504 increased by 8% under normoxic conditions, whereas, for the hypoxic conditions, the increase was 46%. The corresponding values for Pt4 were 10% for normoxic conditions and 34% for hypoxic conditions. Finally, the relative amount of EVs from SKOV3 grown under normoxic conditions decreased by 8% and increased by 28% under hypoxic conditions.

The results displayed in Figure 4 only include one cell concentration. However, the same pattern was also observed for a lower cellular concentration (data not shown). As expected, a higher concentration of cells produced more EVs than a lower concentration of cells, even though a two-fold increase in cell concentration did not result in a two-fold increase in the amount of cell-derived EVs for any of the three cell lines. The influence that the cell concentration had on the EV production was also consistent for all three cell lines tested here (data not shown).

## 4. Discussion

Analysing the protein composition of both EVs and their parent cells is helpful to further understand the mechanism of the EVs' biogenesis and the functional roles that they may play in the development of cancer [41]. Furthermore, the proteomic profile of the cancer-derived EVs is important for discovering novel diagnostic biomarkers and therapeutic targets. Cell culture supernatants have previously been shown to contain EVs [42–44]. Analysing EVs that are released from cell lines from a particular cancer type offers the potential for the identification of EV biomarkers that might be of diagnostic and prognostic value, without the risk of mistaking the EV origin. This is one advantage of using cell lines when looking into cancer research. The EV phenotypes presented here can only reflect the actual cell that they derive from, which makes it easier to interpret the data. In an EV population obtained from a heterogeneous cell population, there are several cells that can act both as producers and recipients of EVs.

Prior to relating specific EV features to cancer or to other pathological conditions, it is of great importance to gain knowledge about EVs in general and in cancer-like conditions.

Several studies prior to this have phenotyped EVs but only with one or a few protein markers and with extensive purification steps, as reviewed in [7]. This has mostly been done with Western blotting [45,46] and flow cytometry [47,48].

The EV Array used for the investigation of the phenotypes and quantification of EVs from the three ovarian cancer cell lines is a microarray technique that was developed in our laboratory. This technique is possible to perform without prior purification of the samples.

In the present setup, the EV Array was optimized to analyse the EV subset that carry the general exosomal markers, CD9, CD63 and CD81, and have a size of approximately 30–100 nm.

Additionally, the EV Array provides an estimated relative quantification of EVs in the samples by measuring the total fluorescence signal when a cocktail of anti-CD9,-CD63 and -CD81 is used to both capture and detect the EVs. Our data indicate that cells from all three cell lines subjected to hypoxic conditions for 24 hours have a tendency to produce more EVs than cells subjected to normoxic conditions, independent of cellular concentration and level of confluence (Figure 3). Nevertheless, with these data, it could not be decided whether a relatively higher amount of EVs or a relatively higher amount of the markers, CD9, CD63 and CD81, were produced under these conditions. However, the measurement here is believed to be a semi-quantitative measurement of the amount of EVs. This is based on the fact that the phenotyping of EVs from both COV504 and Pt4 display an increase in the level of CD9, CD63 and CD81 after 24 hours of cell incubation under normoxic conditions compared to hypoxic conditions (Figure 2). Thereby, the phenotyping displays the opposite situation of the semi-quantification, which points to a higher amount of EVs produced under hypoxia.

This observation was not surprising as it has earlier been shown for rat osteoblasts [42], breast cancer cell lines [43,49] and lung cancer cell lines [44]. Notably, there were more cells after 24 hours of incubation in the cells subjected to normoxic versus hypoxic conditions and, in the case of COV504, even as much as double the amount of cells (Table I). Moreover, the viability of most of the cells was the same for both normoxic and hypoxic conditions. This could indicate that the cells subjected to normoxia mostly focused on proliferation, whereas the cells subjected to hypoxia focused on EV release. This is in-line with another study that reported that breast cancer cells exposed to hypoxia increased their production of microvesicles, which stimulated invasion and metastasis of recipient breast cancer cells [49]. SKOV3 already reached confluence before 24 hours, without altering their viability (see Table I) but, despite this, they continued to produce EVs. Even though these data do not conclude that the level of confluence is without influence on the EV production, they may indicate that cells do not stop producing EVs when they reach confluence.

The fact that more EVs are produced per hour in the early cultures, which are less confluent, might be explained by the fact that cells produce EVs to communicate with each other and the distance between cells are smaller in the cell cultures with high confluence than in the ones with low confluence. Therefore, they need more communicational agents to communicate. It could also be because, as cell lines are copies of themselves, the cells in such cultures only communicate with themselves. Surely, the picture *in vivo* is different and much more complex. This is probably the case as all three cell lines are viable and grow just as well after 12 hours as after 24 hours. The point that more EVs can be measured after 24 hours than after 12 hours only suggests that EVs are not quickly degraded or taken up by the cells. Instead, they stay in the culture for at least 24 hours.

Previously, EVs have been referred to as small copies of the cells that they derive from but more and more evidence show that this is not always the case. Proteins that are highly expressed on EVs are not always expressed by the cells (own observations for CD9, unpublished data). As EVs often reflect the cells that they derive from, it is of interest to see how different the expression levels are between EVs and their parental cells. The data presented in this study clearly show that the EVs are not exact copies of their parental cells, even though the EVs reflect the cells that they derive from. This is also consistent with data from *Im H et al., 2014*, who show a phenotypical comparison of EVs and cells from different ovarian cancer cell lines using flow cytometry and a nano-plasmonic exosome assay [50].

The overall EV phenotype from the three ovarian cancer cell lines show resemblances to each other, especially when comparing the overall EV phenotype to other cancer cell lines (data not shown). Even though all three cell lines show resemblances to each other, they do so with variable degrees. Different conditions influence the cell lines differently and, therefore, must be said to express different EV subsets. The different EV subsets that are seen between the three cell lines might be explained by the fact that the cells that the EVs derive from come from different origins. Furthermore, the different EV subsets that are seen and how they vary due to hypoxic conditions and time could indicate that they have different functions depending on which cell type they derive from. These data clearly show that hypoxia, in particular, has an effect on the expression of proteins on EVs, even though there is no clear correlation between the three cell lines. In particular, SKOV3 is highly affected by hypoxia as it expresses considerably more markers after 24 hours of hypoxic conditions compared to normoxic conditions. Our data are supported by another study that showed that vesicles released under hypoxia, compared to vesicles released under normoxia, were loaded with unique proteins that could enhance invasiveness and induce microenvironment changes of the recipient cells [51]. Furthermore, the expression level of the general EV markers, CD9, CD63 and CD81, on EVs were clearly affected by hypoxia. These data still support CD9 and CD81 as useful general EV markers but it should be noted that the expression level of CD9 and CD81 can be affected by several factors. Other interesting markers are MUC1, EGFR and CD142, which are all highly related to cancer, particularly ovarian cancer. Firstly, many cancers, including ovarian, overexpress MUC1 [52]. EGFR is reported to be both increased in copy number and overexpressed in serious ovarian carcinoma and is associated with a high tumour grade, large residual tumour size, high proliferation index and poor patient outcome [53]. Finally, CD142 regulates tumour cell proliferation and apoptosis, and can promote tumour angiogenesis and metastasis in several different cancers, as reviewed by Han X, et al. [54]. Detected with the EV Array, all three markers were up-regulated in EVs containing supernatant from SKOV3 cells subjected to 24 hours of hypoxia compared to normoxia. The multiplexed protein profiling of EVs provides simultaneous information about numerous possible biomarkers, which increase the power of discrimination [55,56]. Thus, if several of the proteins investigated in the present study were combined, the likelihood of finding biomarkers, which are of clinical relevance for early detection of ovarian cancer, is greater.

In general, when looking at the five markers, CAIX, CAXII, CD151, CD81 and CD9, the cellular phenotype was mostly unaffected by time and oxygen level, whereas their corresponding EVs were much more affected. The only difference in the cellular expression of markers was seen for CD9 on COV504 after 24 hours of either normoxic or hypoxic conditions. This might be explained by the fact that the hypoxic cell population had a considerable decreased viability compared to the normoxic cell population. The phenotyical differences seen between the cells and their corresponding EVs cannot be explained by only one of the factors (time, cellular concentration or oxygen level) but rather, a combination of them. Even though there were more EVs in the highest cellular concentration in the hypoxic population after 24 hours, both COV504 and Pt4 displayed an increase in the level of most markers in the normoxic population, whereas EVs from SKOV3 showed the opposite. This might support the idea that EVs are used by cells for fast and effective communication with their surroundings. One study has even reported that EVs can deliver a biologically active transcriptional factor, Hif-1α, to a recipient cancer cell line [57].

To further stress the importance of investigating EVs and their phenotypes for their potential role in cancer, a recently published study showed that plasma exosomes from cancer cells present a tumour-related proteomic profile. Furthermore, the levels of bioactive molecules contained in the exosomes of patients with solid tumours before surgery are significantly higher than in exosomes from the same patients after surgery [58].

Learning more about the role that EVs play in cellular communication in healthy, and particularly under pathological conditions, will hopefully help to understand the way that cancers develop and metastasize. The tumour milieu has long been recognized as being important in the way that cancers develop. Others have also shown that pH and hypoxia can influence cell migration [35,49,51]. The presented data emphasize the complexity of factors that might influence the cellular and EV phenotype and thereby the metastatic process.
